# Effect of body mass index on symptomatic venous thromboembolism and prosthesis revision risk after total knee arthroplasty: a long-term study from China

**DOI:** 10.1186/s12891-022-05634-w

**Published:** 2022-07-15

**Authors:** Changjie Shao, Kuishuai Xu, Liang Zhang, Tengbo Yu, Ning Yu

**Affiliations:** 1 Department of Ultrasound, Affiliated Hospital of Qingdao University, Qingdao, 266000 Shandong China; 2 Department of Orthopaedic Surgery, Affiliated Hospital of Qingdao University, Qingdao, 266000 Shandong China

**Keywords:** Body mass index, Total knee arthroplasty, Venous thromboembolism, Revision

## Abstract

**Objective:**

To investigate the effect of body mass index (BMI) on the risk of symptomatic VTE and prosthesis revision after total knee arthroplasty (TKA).

**Methods:**

Seven thousand one hundred eighty-two patients with primary unilateral TKA treated in our hospital from 2011 to 2020 were divided into four groups according to BMI: BMI < 25 kg/m^2^, BMI 25 kg/m^2^–29.9 kg/m^2^, BMI 30 kg/m^2^–34.9 kg/m^2^ and BMI ≥ 35 kg/m^2^. Incidence, Odds ratio and Kaplan-Meier survival analysis were used to evaluate the effects of BMI on symptomatic VTE and prosthesis revision risk after TKA.

**Results:**

The incidence of VTE after TKA was 8.9‰(64/7182). There was no significant difference in the incidence of VTE among different BMI groups(*P* = 0.452). Deep vein thrombosis mainly occurred in the distal lower extremities, especially in intermuscular veins. Revision rate of prosthesis after TKA was 6.4‰(46/7182). There was no significant difference in revision rate among different BMI groups(*P* = 0.718). In the univariate analysis of TKA, compared with patients with normal BMI, the risk of postoperative VTE and prosthesis revision in patients with overweight, obesity class I and obesity class II did not increase. Higher prosthesis revision rate and lower prosthesis survival rate were observed in BMI ≥ 35 kg/m^2^ group, although the difference was not statistically significant.

**Conclusions:**

Through such a retrospective large sample data of long-term follow-up, we believe that the higher BMI was not associated with the increased risk of symptomatic VTE and prosthesis revision after TKA. When TKA was used for appropriate indications, high BMI should not be considered as a contraindication.

## Introduction

Obesity [1, 2] is recognized as a major risk factor for knee osteoarthritis, so the effect of obesity on surgical outcomes is also of interest to clinicians and patients. As obesity continues to rise, so does the need for knee arthroplasty. TKA is a reliable treatment for patients in the middle and late stages of knee osteoarthritis, and in obese patients who undergo TKA postoperatively, pain is significantly reduced and functional recovery is good [3, 4]. Nonetheless, several studies have noted that obesity increases the rate of perioperative complications after TKA, such as increased overall revision surgery rates due to infection [5–9], and increased rates of VTE events (defined as deep vein thrombosis (DVT) or pulmonary embolism (PE)) [10–12]. However, part of the studies were limited by sample size and the results need to be interpreted with caution.

Some recent studies [13–17] have expressed the opposite view that there is no association between BMI and the occurrence of VTE events after TKA and the increased risk of prosthesis revision. Crookes et al. [16] showed that there was no significant difference in VTE events between patients with morbidly obese (BMI ≥40 kg/m^2^) and patients with BMI < 40 kg/m^2^. Tang et al. [17] divided the patients into 5 groups according to BMI and found that there was no statistical difference in the incidence of total VTE among different BMI groups. Molloy et al. [13] conducted a prospective study over a period of 10 years, and the results showed that there was no difference in the survival rate of implant survival among different BMI groups. A multicenter retrospective study conducted by Affatato et al. [14] found that obese and morbidly obese patients benefited as much from total knee arthroplasty as non-obese patients, and no statistical difference in prosthesis survival was observed. Another meta-analysis [15] concluded that the mean revision rate in obese patients (BMI>30 kg/m^2^) was 0.33% pa (95%CI-3.16 to 2.5) higher than in non-obese patients, however this was not statistically significant (*p* = 0.82).

Therefore, it is not clear whether patients with elevated BMI are more likely to develop VTE and have a higher prosthesis revision rate than patients with normal BMI, and as far as we know, few studies have analyzed the incidence of thrombosis and prosthesis revision rate in patients after TKA. The purpose of this study includes two aspects: (1) To evaluate whether the increase of BMI will increase the risk of symptomatic VTE and prosthesis revision after TKA. (2) The distribution and imaging characteristics of lower extremity deep venous thrombosis were recorded, and the reasons for prosthesis revision were descriptively analyzed.

## Materials and methods

### Patient selection

This study is a retrospective study. After the approval of our institutional review committee (QYFL WZLL 26879), we searched the clinical data of 11,514 patients who underwent TKA from January 2011 to December 2020 in our hospital. Inclusion criteria: 1) Meet the clinical diagnosis of knee osteoarthritis; (2) All patients underwent primary unilateral TKA; (3) No rheumatism/inflammatory arthritis. Exclusion criteria: (1) Previous history of thrombosis or preoperative diagnosis of lower extremity DVT; (2) Patients with recent history of fracture or bone tumor; (3) Incomplete clinical data or loss of follow-up; Finally, 7182 patients were included in our cohort study. A detailed case review was conducted for each patient, and the collected clinical data included; Gender, age, affected side, operation time, length of stay and number of combined diseases. At the same time, record the patient’s height and weight, and calculate the BMI (weight divided by the square of the height).

### Surgical technique and data collection

The operation of all patients was completed by the experienced chief physician of our hospital. Tourniquet was used throughout the operation. All patients were treated with medial approach. Patellar replacement was not routinely performed. The femoral prosthesis and tibial prosthesis was fixed with bone cement. Polyethylene spacer was installed. The thumbless test, knee joint stability and good range of motion were examined.

All patients were followed-up by outpatient, medical record system and telephone to determine the incidence of VTE events and prosthesis revision after TKA, and the causes of revision were descriptive statistics. The diagnostic indicators of VTE include: (1) clinical manifestations: persistent pain, swelling, local deep tenderness, pain in posterior flexion and restricted movement of the lower limbs and other discomfort, DVT of the lower limbs should be suspected; Postoperative unexplained shortness of breath, dyspnea, chest pain, syncope, hypotensive shock should be suspected as PE. (2) Laboratory tests showed elevated D-dimer. In case of the above, color Doppler ultrasound was immediately performed. If PE was suspected, the diagnosis was confirmed by transpulmonary CT angiography. We also recorded the location of the thrombus, thrombus echogenicity (hyperechoic, hypoechoic), number of involved vessels, and total thrombus length.

### Exposure

Patients were classified into four sub-groups a priori based on BMI at the time of surgery. Groups were BMI < 25 kg/m^2^(normal), BMI 25 kg/m^2^–29.9 kg/m^2^(overweight), BMI30 kg/m^2^–34.9 kg/m^2^ (obesity class I) and BMI ≥ 35 kg/m^2^ (obesity class II), and are consistent with the classifications of obesity defined by the World Health Organization.

### Statistical analysis

Data analysis was performed using SPSS 25.0 statistical software. The measurement data with normal distribution and homogeneous variance are expressed by mean ± standard deviation. The independent sample t-test is used for the comparison between groups, and the non-parametric test is used when the distribution is skewed or the variance is uneven. Enumeration data were expressed in frequency or percentage, and chi-square test was used for comparison between groups. Univariate logistic regression was used to compare the predictive value of obesity category on the incidence of symptomatic VTE and prosthesis revision. The normal weight (BMI < 25 kg/m^2^) category was used as the control group. Kaplan Meier survival curve was drawn and the revised data were analyzed. Statistical significance was defined as *p* value< 0.05.

## Result

### Patients

During the study period, 11,514 patients with knee osteoarthritis were evaluated. According to the exclusion criteria, 7182 patients were enrolled in the study cohort, including 2264 males and 4918 females, with an average age of 65.76 ± 7.2 years (range 23–87 years). BMI < 25 kg/m^2^ group 2036 cases (28.3%), BMI 25–29.9 kg/m^2^ group 3691 cases (51.4%), BMI 30–34.9 kg/m^2^ group 1279 cases (17.8%) and 176 cases (2.5%) in BMI ≥ 35 kg/m^2^ group. The increase in BMI was associated with lower age, higher proportion of women, longer surgery and length of stay, more complications and higher hospitalization costs, there was no statistically significant difference between the groups in terms of surgical side (Table 1).

**Table 1:** Baseline characteristics according to four body mass index (BMI) categories

	BMI<25 (*n* = 2036)	BMI25–29.9 (*n* = 3691)	BMI30–34.9 (*n* = 1279)	BMI ≥ 35 (*n* = 176)	*P*
Age, mean ± SD	66.6 ± 8.2	66.0 ± 7.3	64.9 ± 7.1	63.8 ± 8.2	<0.001
Sex					0.003
Women	1358	2595	840	125	
Men	678	1096	439	51	
Affected side					0.289
Left	993	1821	591	86	
Right	1043	1870	688	90	
Length of stay	9.2 ± 3.6	9.2 ± 4.3	9.4 ± 3.8	9.9 ± 4.1	0.031
Surgery time, mins	83.2 ± 25.7	83.7 ± 25.8	85.2 ± 24.6	89.9 ± 25.1	<0.001
Total expense, RMB	49,141.8 ± 17,126.6	57,477.6 ± 22,957.5	58,515.8 ± 16,421.8	71,234.9 ± 25,902.8	<0.001
BMI, mean ± SD	22.9 ± 1.7	27.3 ± 1.4	31.8 ± 1.4	38.1 ± 3.9	<0.001
Number of combined underlying diseases					<0.001
0	715	1151	359	53	
1	683	1287	447	60	
≥ 2	638	1253	473	63	

### Outcome measures

The total incidence of VTE events after TKA was 8.9‰ (64/7182), including 7.9‰ (16/2036) in BMI < 25 kg/m^2^ group, 10.6‰ (39/3691) in BMI 25–29.9 kg/m^2^ group, 6.3‰ (8/1279) in BMI 30–34.9 kg/m^2^ group and 5.7‰ (1/176) in BMI ≥ 35 kg/m^2^ (Table 2). From Table 3, it is not difficult to find that most deep venous thrombosis mainly occurs in the distal lower extremities, especially in the intermuscular veins, as high as 87.5% (56/64). There was no statistical difference in thrombus echo, thrombus length and the number of blood vessels involved among different BMI groups (*P*>0.05) (Table 4).

**Table 2:** Incidence of VTE and prosthesis revision among different obese categories

	VTE(*n* = 64)	Revision(*n* = 46)
BMI<25	16 (25.0%)	14 (30.4%)
BMI25–29.9	39 (60.9%)	24 (52.2%)
BMI30–34.9	8 (12.5%)	6 (13.1%)
BMI>35	1 (1.6%)	2 (4.3%)
X2	2.630	1.347
P	0.452	0.718

Data on percentage are number of cases/total VTE cases or total revised cases

**Table 3:** Comparison of distribution of deep venous thrombosis among different obese categories

Proximal	Case
CFV	4
DFV	2
SFV	6
PV	9
Distal	
PTV	7
PeV	2
MV	56

*CFV* Common femoral vein, *DFV* Deep femoral vein, *SFV* Superficial femoral vein, *PV* popliteal vein, *PTV* Posterior tibial vein, *PeV* Peroneal vein, *MV* Muscular vein

**Table 4:** Comparison of imaging characteristics of lower limb vascular ultrasound among different obese categories

	BMI<25	BMI25–29.9	BMI30–34.9	BMI>35	P
Number of embolized vessels					0.335
1	9	21	6	0	
2	2	10	0	1	
≥ 3	5	8	2	0	
Echogenicity					0.072
Hypoechoic	16	37	8	0	
Hyperechoic	0	2	0	1	
Mean vein diameter (mm)	50.3 ± 29.1	46.7 ± 27.6	42.6 ± 13.6	56.0	0.910

**Fig. 1:**
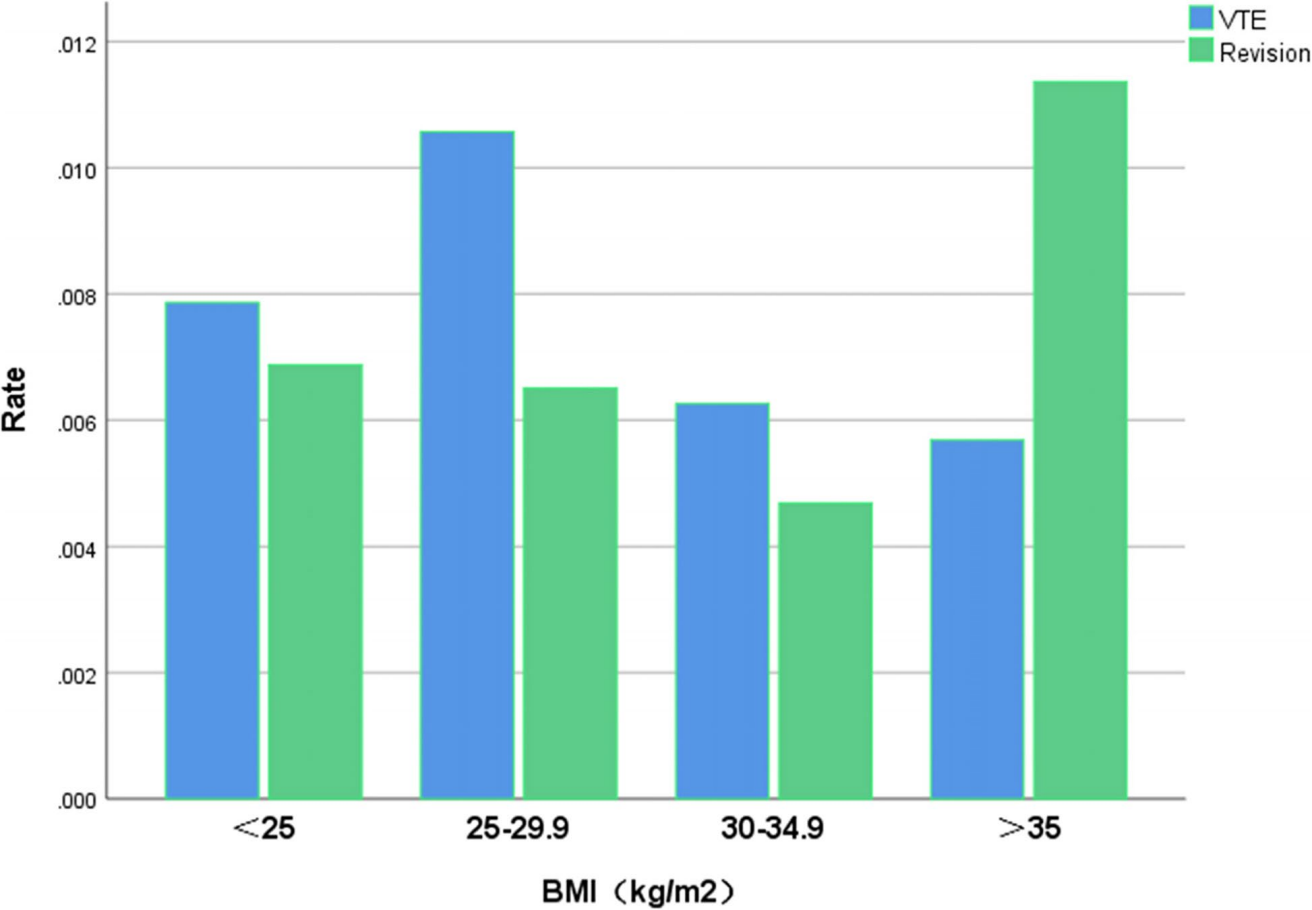
Bar graph illustration of VTE and revision rate among all BMI groups

A total of 46 revisions were made throughout the study period, with a total revision rate of 6.4‰ (46/7182). The revision rate was 6.9‰ (14/2036) in BMI < 25 kg/m^2^ group, 6.5‰ (24/3691) in BMI 25–29.9 kg/m^2^ group, 4.7‰ (6/1279) in BMI 30–34.9 kg/m^2^ group and 11.4‰ (2/176) in BMI ≥ 35 kg/m^2^ group. Patients with BMI ≥ 35 kg/m^2^ had a relatively higher risk of prosthesis revision (Fig. 1), although there was no significant difference in prosthesis revision rate among different BMI groups (*P* = 0.718) (Table 2). In the univariate analysis of TKA, compared with patients with normal BMI, the risk of postoperative VTE and prosthesis revision in patients with overweight, obesity class I and obesity class II did not increase (Table 5).

**Table 5:** Results of univariate regression models comparing the outcomes of VTE and revisions among different obese categories

Groups	BMI25–29.9 vs. BMI<25		BMI30–34.9 vs. BMI<25		BMI ≥ 35 vs. BMI<25	
	OR(95%CI)	*P*	OR(95%CI)	*P*	OR(95%CI)	*P*
VTE	1.35(0.75–2.42)	0.316	0.79(0.34–1.86)	0.595	0.72(0.10–5.47)	0.752
Revisions	0.95(0.49–1.83)	0.868	0.68(0.26–1.77)	0.431	1.66(0.37–7.36)	0.505

**Fig. 2:**
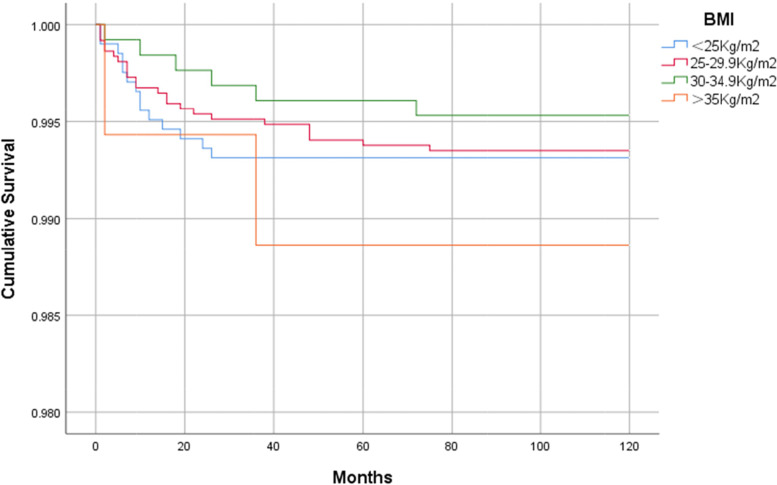
Survival curve of Kaplan–Meier prosthesis in four body mass index (BMI) categories

With regard to the causes of prosthesis revision: In 46 patients with revision, our data showed that infection was the main cause of revision (26/46), followed by aseptic loosening (9/46) (Table 6). Kaplan-Meier survival analysis showed that: graphically, survival did not appear to be different between BMI < 25 kg/m^2^, BMI 25–29.9 kg/m^2^ and BMI 30–34.9 kg/m^2^, and the group with BMI ≥ 35 kg/m^2^ had a relatively low prosthesis survival rate, with a 10-year survival rate of 98.9%, but the difference was not statistically significant (Fig. 2).

**Table 6:** Reason for revision and revision rate

Reason	Case(*n* = 46)	Rate(%)
Periprosthetic joint infection	26	56.5
Aseptic loosening	9	19.6
Pain without loosening	4	8.7
Periprosthetic Fracture	2	4.3
Knee instability	2	4.3
Dislocation	1	2.2
Liner wear	1	2.2
Stiffness	1	2.2

Data on percentage are number of cases/total revised cases

## Discussion

The occurrence of VTE events after TKA can lead to serious outcomes, including high mortality, prolonged hospitalization and increased hospitalization costs, etc. [18], and the revision of postoperative prosthesis is more a psychological and economic burden to patients. Therefore, preventing the occurrence of VTE events and prosthesis revision remain important tasks for quality improvement programs after TKA. But current literature is still conflicting about the effect of obesity on symptomatic VTE and prosthetic revision after TKA, and part of the literature is affected by small sample size or the rarity of VTE events. This study therefore sought to address this major question: whether BMI is associated with an increased risk of VTE events and prosthetic revision after TKA.

With regard to the risk of VTE after TKA in different BMI populations, Mantilla et al. [12] conducted a retrospective case-control study to determine the clinically related risk factors of DVT and PE after TKA, they found that obesity defined as BMI > 30 kg/m^2^ imparted a 3.4 times elevated risk of VTE compared with controls. In addition, White et al. [19] concluded that BMI ≥ 25 kg/m^2^ was associated with subsequent hospitalization for thromboembolism when evaluating VTE risk factors for re-hospitalization after TKA. However, Friedman et al. [20] found that morbid obesity is only associated with an increase in early postoperative complications, not with an increased risk of VTE or bleeding. Cafri et al. [21] conducted a study followed-up for 10 to 18 years, the results showed that there was no statistically significant difference in DVT between obese and non-obese patients after TKA. Tang et al. [17] showed that the total incidence of VTE in different BMI groups was statistically similar. However, the sample size in some of the above studies is relatively small and may not be sufficient to detect differences between groups. The advantages of our study are large sample size, long follow-up time, and detailed description of the incidence of DVT, ultrasonographic features and thrombus distribution in different BMI groups. Rigorous statistical analysis of our data shows that there is no correlation between the increase in BMI and the increased risk of VTE events after TKA. This is consistent with the results of Sloan et al. [22] Sloan and colleagues reviewed the American College of Surgeons National Surgical Quality Improvement Program (ACS-NSQIP) database, which included 218,997 patients with initial TKA and 15,286 patients with revised TKA, and concluded that patient classification as overweight or obese is associated with increased risk of development of PE but not DVT after TKA. Xu et al. [23] conducted a nested case-control study based on a large data set of 15,326 patients. The results showed that BMI was not a risk factor for DVT after TKA. This report also confirms our point of view.

In this study, the incidence of VTE events after TKA was 0.89%, which was lower than that reported in previous studies [24, 25]. However, similar to the results found in the Asian population, Tay et al. [26] reported a low prevalence of VTE in their study population (0.67%); A cohort study based on the Taiwan population found that the total incidence of VTE after TKA was 0.64% [27]. The difference in incidence may be due to differences in race, number of subjects, follow-up time and use of anticoagulants. As this systematic review [28] concludes, black race as a risk factor for VTE for TKA, while Hispanic race showed no significant difference when compared to the white race. In our study, the accuracy of lower limb vascular ultrasound in the diagnosis of DVT may be lower than that of lower limb deep venography, but it has the advantages of non-invasive and convenient. In addition, lower limb vascular ultrasound can also improve a lot of thrombus-related information for us, including thrombus location, thrombus echo and thrombus length, so that we can carry out statistical analysis. Our data suggest that deep venous thrombosis occurs mainly in the calf intermuscular vein (56/64), and the echo of thrombus is mainly hypoechoic. No statistical differences were observed in thrombus length, thrombus echo and the number of blood vessels involved among different BMI groups, but a larger sample size is needed for more in-depth study in the future to verify our conclusions.

Regarding the risk of prosthetic revision after TKA in different BMI populations, it has been a hot spot of clinical research and is also widely debated. Previous studies [29] have shown that prosthetic revision rates are similar in patients with a BMI < 35 kg/m^2^, but are significantly increased at a BMI ≥ 35 kg/m^2^. Electricwala et al. [30] suggested that an elevated BMI is a risk factor for early referral to a tertiary care center for revision TKA. However, some studies [31–33] reported the opposite conclusion. Ayyar and colleagues [32] did not observe a difference in the number of revisions between different BMI levels, and they suggested that surgery provides a similar degree of benefit regardless of the patient’s BMI level. Gaillard et al. [33] investigated the long-term survival of the prosthesis using Kaplan-Meier analysis and showed that obesity had no effect on the medium-term prosthesis survival. The present study confirms the latter statement. Our study showed that no statistical difference was found in the risk of prosthetic revision between different BMI groups, and we believe that a higher BMI should not be considered a risk factor for revision for mechanical purposes.

We also found no significant difference in the 10-year survival rates of prosthetic in different BMI groups, and although patients with a BMI ≥ 35 kg/m^2^ had relatively poor survival rates, the differences were not statistically significant, and our findings are supported by a recent study [34]. On the other hand, the results shown in our data are encouraging, with 10-year survival rates of 98% or higher for all BMI groups. Interestingly, patients in the overweight and obese class I had the highest 10-year survival and those in the normal BMI range had the worst survival, this is counter-intuitive, as most people tend to associate healthy weight range with increased prosthetic survival. However, this difference is not obvious, and whether there is a universal law remains to be further studied. In addition, descriptive statistics were also performed for the reasons for revision in our study, which showed that infection was the leading cause of revision (26/46), followed by aseptic loosening (9/46), which was consistent with the results of several previous studies reporting the causes of prosthetic revision [30, 35, 36]. Bozic et al. [35] reported that the most common reasons for revision TKA were infection (25.2%) and implant loosening (16.1%). Hossain et al. [36] reported that common causes of revision TKA included infection (32.7%), aseptic loosening (14.9%), and polyethylene wear (12.3%). In summary, periprosthetic infection is still the most common cause of revision and re-revision TKA.

Our research also has some limitations. First, this is a retrospective study conducted in a single institution. This may limit the external effectiveness of our study, and multicenter studies are needed to further verify our conclusions; Second, some patients may have undergone revision surgery in another hospital without reporting this information to us. However, as far as we know, this situation is rare. Third, the study could not comment on BMI-wide extremes. There were not many patients in our cohort with class III obesity (BMI ≥ 40 kg/m^2^) only 34 patients. Finally, some patients have a short follow-up period, which may be considered a limitation for patients with shorter follow-up time, considering that aseptic prosthesis loosening may occur during long-term follow-up. Therefore, in the context of these restrictions, our results must be interpreted carefully.

## Conclusion

In this study, we concluded that patients with BMI ≥ 35 kg/m^2^ had a relatively high revision risk, although the difference was not statistically significant, and that higher BMI was not related to the increased risk of VTE events and prosthesis revision after TKA. Based on these findings, we believe that high BMI should not be regarded as a surgical contraindication when TKA is used for appropriate indications.

## Data Availability

The datasets generated during and/or analyzed during the current study are available from the corresponding author on reasonable request.

## Abbreviations

TKA: Total knee arthroplasty
VTE: Venous thromboembolism
BMI: Body mass index
